# Retraction: Revisiting hydrocephalus as a model to study brain resilience

**DOI:** 10.3389/fnhum.2016.00375

**Published:** 2016-07-22

**Authors:** Human Neuroscience Editorial Office

The journal retracts the 6 January 2012 article cited above. Following a series of concerns regarding the origin of images in this article, Frontiers conducted an investigation. The results of this investigation determined that, as these images formed an integral part of the article and did not originate in the authors' laboratories and were not duly attributed, the article does not meet the scientific criteria of the journal. This retraction was approved by the Specialty Chief Editors of *Frontiers in Human Neuroscience*. The authors concur with the retraction and sincerely regret any inconvenience this may have caused to the reviewers, editors, and readers of *Frontiers in Human Neuroscience*.

